# Production and application of peptidyl-lys metalloendopeptidase: advances, challenges, and future perspectives

**DOI:** 10.1007/s00253-025-13473-7

**Published:** 2025-04-10

**Authors:** Uzair Ahmed, Katrin Ochsenreither, Thomas Eisele

**Affiliations:** 1 Faculty of Mechanical and Process Engineering, Hochschule Offenburg, 77652 Offenburg, Germany; 2 Department of Chemical and Process Engineering, Karlsruhe Institute of Technology (KIT), 76131 Karlsruhe, Germany

**Keywords:** Peptidyl-lys metalloendopeptidase, MEP, Lys-N, Trypsin, Proteomics, Mass spectrometry

## Abstract

Peptidyl-lys metalloendopeptidases (PKMs) are enzymes that selectively cleave peptide bonds at the N-terminus of lysine residues present in the P1′ position, making them valuable tools in proteomics. This mini-review presents an overview of PKMs, covering their traditional production from native sources, recent advances in recombinant production, and the current limitations in availability. The historical and current applications of PKMs in proteomics are discussed, highlighting their role in protein sequencing, peptide mapping, and mass spectrometry-based studies. Advances in recombinant technology now enable tailored modifications to PKM, allowing it to function not only as a sister enzyme to LysC but also to trypsin, thereby enhancing its suitability for specific analytical applications. The mini-review concludes with a forward-looking statement on PKM research, emphasizing the potential to broaden its use in novel proteomic methods and other applications.

## Introduction

**Fig. 1:**
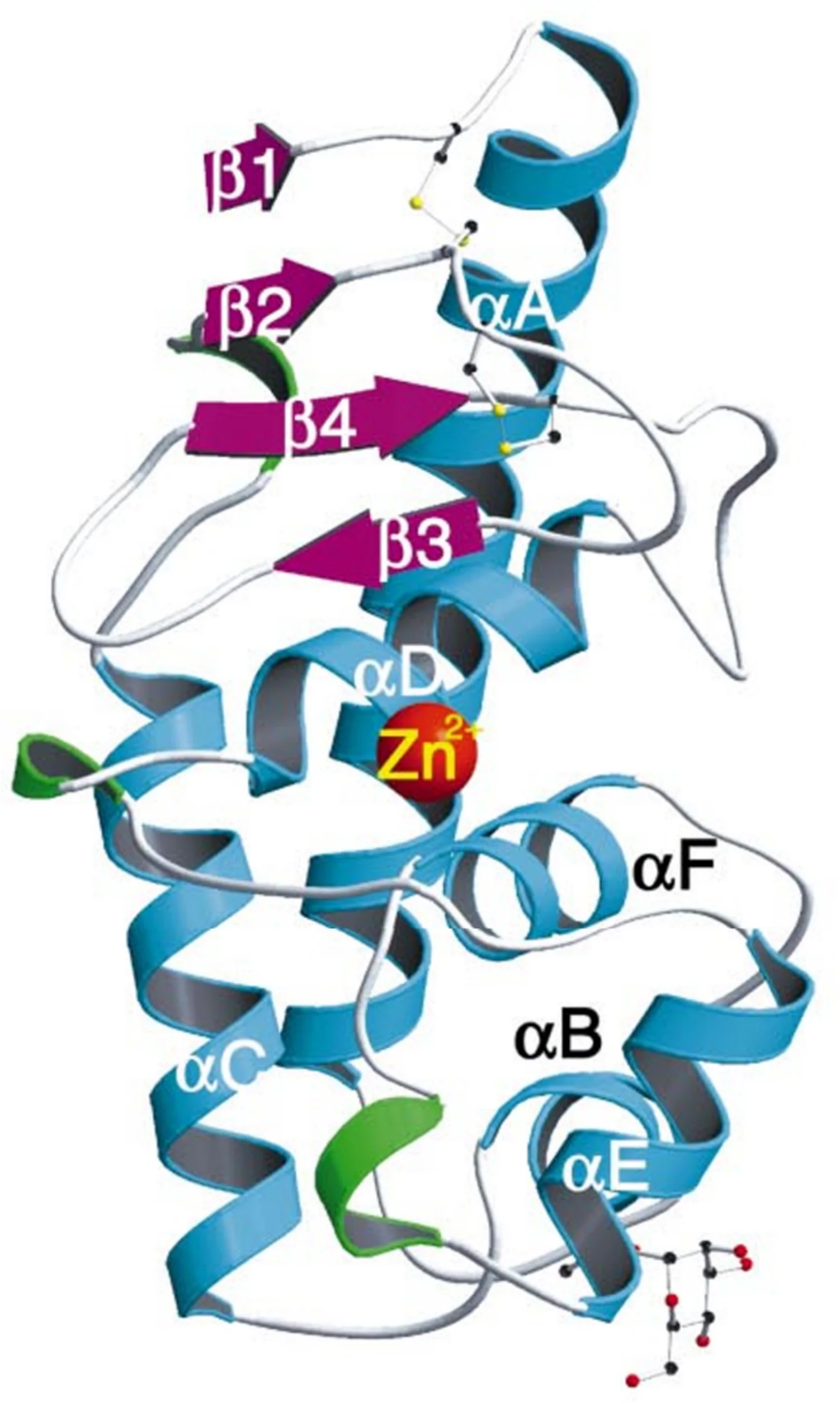
Ribbon model of the PKM from *G. frondosa* (Hori et al. 2001; reproduced with permission from the International Union of Crystallography)*.* α-Helices represented in cyan, β-strands in magenta, β-turns in green, and loops in grey. The catalytic zinc ion, two disulfide bridges, and Thr42 with O-linked mannose are illustrated as ball-and-stick models. Colors of atoms are as follows: carbon in black, oxygen in red, sulfur in yellow, and zinc in orange.

Peptidyl-lys metalloendopeptidases (PKMs; E.C. 3.4.24.20), also known as LysN (or Lys-N) peptidases or MEPs, are a class of proteolytic enzymes that selectively cleave N-terminus peptide bonds at lysine (K) residues present in the P1′ position (Raijmakers et al. 2010). PKMs are placed in the MEROPS peptidase clan “MA (D)” and belong to the deuterolysin metallopeptidase family “M35” (M35.004; Rawlings and Salvesen 2013). The divalent metal ion, zinc, plays a crucial role as co-factor in the catalytic activity of PKMs. The tertiary structure of PKMs comprises five alpha helices and four beta strands. It has been reported that a conserved tyrosine, located three residues downstream from the zinc-binding aspartate, functions as a proton donor during the catalytic process (Hori et al. 2001). One zinc atom per enzyme molecule is an essential component and proteolytic activity is inhibited by chelating agents such as EDTA and 1,10-phenanthroline (Rawlings and Salvesen 2013). The molecular weight (MW) of mature (active) PKMs typically ranges between 16 and 20 kDa. Structure of a representative PKM from *Grifola frondosa* illustrated by Hori et al. (2001) is depicted in Figure 1.

Due to its cleavage specificity for lysine residues present in the P1′ position and a MW smaller than that of trypsin, PKM is often evaluated in comparison to lysyl endopeptidase, also known as LysC (or Lys-C) peptidase, and trypsin (Tsiatsiani and Heck 2015). Table 1 highlights the key differences between the three peptidases. PKM and LysC are closely related but differ in their cleavage specificities, with PKM targeting the N-terminus of lysine (P1′), while LysC cleaves at the C-terminus of lysine (P1). Trypsin, however, cleaves at the C-terminus of both lysine and arginine residues present in the P1 position, resulting in shorter peptide generation. In contrast, PKM and LysC provide more selective cleavage at lysine, making them valuable alternatives for specific applications where finer control over peptide generation is needed. A defining physiochemical feature of PKMs reported so far is their resistance to high concentrations of denaturants such as urea (≤ 8 M) and guanidine hydrochloride (≤ 1 M). Since these denaturants are commonly used in mass spectrometry (MS) analyses, the most notable applications of PKMs have focused on MS protocols, which are discussed later in this mini-review.

**Table 1:** Comparison of key features of PKM, LysC, and trypsin

**Peptidase**	**Common names(s)**	**Enzyme class**	**Source**	**Clan**	**Specificity**	**EC number**
Peptidyl-lys metalloendopeptidase	LysN, Lys-N, PKM	Metalloendopeptidase	*Grifola, frondosa, Armillaria mellea, Pleurotus ostreatus, Trametes coccinea*	MA	P1′: K	EC 3.4.24.20
Trypsin	Trypsin	Serine peptidase	*Bos Taurus, Sus scrofa*	PA	P1: K, R	EC 3.4.21.4
Lysyl endopeptidase	LysC, Lys-C	Serine peptidase	*Lysobacter enzymogene, Pseudomonas aeruginosa*	PA	P1: K	EC 3.4.21.50

Up until recently, any substantial amount of PKMs had been isolated exclusively from the fruiting bodies of higher basidiomycetes, such as *Grifola frondosa*, *Pleurotus ostreatus*, and *Armillaria mellea* (Lewis, Basford, and Walton 1978; Nonaka et al. 1995, 1997). The only exception had been the PKM extracted from the culture supernatant of *Myxobacter* AL- 1 (Wingard, Matsueda, and Wolfe 1972). The first PKMs were extracted as crude preparations of the native enzyme with varying levels of purity (Dohmae et al. 1995; Lewis, Basford, and Walton 1978; Nonaka et al. 1995; Wingard, Matsueda, and Wolfe 1972). These early reports established that PKMs possess a pH optimum that ranges from neutral to alkaline (pH 7.0—9.5). These peptidases were also found to be thermostable even at temperatures as high as 80 °C for up to 3 h (Ahmed et al. 2024; Nonaka et al. 1995).

Recent advances in biotechnology have led to improvements in the production of PKMs via recombinant protein engineering methods. To the best of our knowledge, only one native PKM, derived from the fruiting bodies of *G. frondosa*, and only two recombinant PKMs, derived from *G. frondosa* and an unknown source organism, were ever made commercially available. However, according to our research for this mini-review, the native PKM from *G. frondosa*, sold under the brand name “Pierce™ LysN Protease (MS Grade)”, manufactured by Thermofisher Scientific, and the recombinant “Lys-N” manufactured by U-Protein Express (Utrecht, The Netherlands) are no longer available for purchase. “Lys-N”, recombinantly produced in *Komagataella phaffi* (source organism undisclosed; however, according to manufacturer, it is *not* derived from *G. frondosa*), is currently the only PKM available as a commercial preparation. It is manufactured by ImmunoPrecise Antibodies (Utrecht, The Netherlands) and sold for >€10,000/mg (SKU: L101 - 0.5 mg). This limited availability underscores the opportunity for more research and development to produce cheaper PKMs that can be exploited for research and industrial use. PKMs from *G. frondosa* (Saito et al. 2002), *A. mellea* (Ødum et al. 2016), and *Trametes coccinea* (Ahmed et al. 2024) have been recombinantly expressed by in *K. phaffii*—both as zymogens and as active enzymes. The recently studied peptidase from *Trametes coccinea* was the first PKM to be described as having an acidic pH optimum (pH 5.0).

PKMs and their biochemical characteristics are briefly discussed in the book “*Handbook of Proteolytic Enzymes*”; however, the cited literature is limited to publications up to 2010 (Rawlings and Salvesen 2013). At the time of writing this mini-review, no previous reviews or mini-reviews could be found that explicitly discuss PKM production strategies and their applications. By highlighting developments in these two areas, this mini-review fills a gap in the literature, providing insights into the state-of-the-art in PKM research.

## Production and purification of peptidyl-lys metalloendopeptidase

The discovery of peptidyl-lys metalloendopeptidases marked a significant step in the study of proteolytic enzymes, with their unique ability to selectively cleave peptide bonds at lysine (K) residues present in P1′ position (Raijmakers et al. 2010). Traditionally, these enzymes were sourced from natural organisms, often involving complex extraction processes from microbial cultures or fungal species.

### *Traditional methods*

The first metalloendopeptidase with cleavage specificity for lysine in P1′ position, named “*Myxobacter* AL-1 Protease II,” was purified from *Myxobacter* AL-1’s fermentation broth. This process involved precipitation, resuspension, and column chromatography. The peptidase's lysine specificity was confirmed via hydrolysis of insulin B-chain, cytochrome c, lysozyme, and vasopressin (Wingard, Matsueda, and Wolfe 1972). Since then, lysine-specific (P1′) metalloendopeptidases (PKMs) have been predominantly extracted from fungi known as basidiomycetes. From 1978 to 2000, PKMs were purified from the fruiting bodies of *Grifola frondosa*, *Armillaria mellea*, and *Pleurotus ostreatus*. The purification protocols generally involved homogenization, precipitation (using ethanol or ammonium sulfate), and multiple chromatography steps. Differences in methods included precipitation duration (ranging from 5 to 44 h) and the type of chromatography used. Notable studies include the following:

1. Purification of PKM from *A. mellea*: Lewis et al. (1978) used ethanol precipitation and four chromatography steps; Healy et al. (1999) used similar methods with shorter precipitation times.
2. Purification of PKM from *G. frondosa*: Nonaka et al. (1995) employed ammonium sulfate precipitation (~ 18 h); Stressler et al. (2014) and Zhao et al. (2020) applied advanced chromatography, with the latter utilizing dried fruiting bodies for long-term storage.
3. Purification of PKM from *P. ostreatus*: Dohmae et al. (1995) used prolonged ammonium sulfate precipitation (~ 44 h) followed by five chromatography steps.

The purification highlights outlined above are summarized in Table 2. The content of PKM present inside the fruiting bodies of basidiomycetes typically ranges between 0 and 3% of the total soluble protein content (Dohmae et al. 1995). Lewis et al. (1978) purified 30 mg of PKM from a starting protein content of ≈41 g with a final total activity yield of 35%. However, as noted by Healy et al. (1999), there can be tremendous batch-to-batch variation in the content of PKM found inside the fruiting bodies. According to their report, only 30% batches of collected fruiting bodies of *A. mellea* had any substantial amount of PKM activity. Besides PKM, the fruiting bodies of basidiomycetes contain a multitude of other proteolytic enzymes. More than four endopeptidases and aminopeptidases were detected in the crude preparation of *G. frondosa*’s fruiting bodies (Nishiwaki, Asano, and Ohyama 2009). This abundance of non-specific proteolytic enzymes presents a challenge in determining the actual yield of PKMs when synthetic substrates like azocoll and azocasein are utilized. Since non-target peptidases also hydrolyze these synthetic substrates, initial PKM activity in crude extracts and in subsequent chromatography fractions cannot be accurately quantified. Stressler et al. (2014) solved this problem by designing a PKM-specific HPLC-based enzyme assay to estimate the yield of pure PKM. The novel method took advantage of PKMs’ high cleavage specificity towards the amino terminal lysine residue. PKM-specific enzyme assay utilized hippuryl-Lys-Val-OH (hip-KV) as substrate. After hydrolysis by the PKM, the enzymatic reaction mixture was analyzed via RP-HPLC. PKM activity was determined by detecting (at 228 nm) and quantifying the liberated hippuric acid. According to their report, the final total activity yield of the purified PKM from *G. frondosa* was 31% when the PKM-specific enzyme activity assay was employed vs the final total activity yield of only 0.21% when the nonspecific azocasein assay was employed. The yield determined for the partially purified PKM (assessed on PAGE) with the PKM-specific assay was in congruence with the yields of PKMs reported in two of the studies discussed above (35% and 25%). This could indicate that there might have been low concentrations of non-target peptidases present inside the crude preparations from which the PKMs in these studies were purified. This observation is also in agreement with the findings of Dohmae et al. (1995) and Healy et al. (1999).

**Table 2:** Summary of key purification parameters during isolation of PKMs via traditional extraction methods

**Organism**	**Starting material; mass or volume**	**Extraction or precipitation medium**	**Purification steps**	**Purity**	**Final total activity yield [%]**	**Enzyme assay substrate**	**Reference**
*Myxobacter* AL-1	Fermentation broth; 112 liters	Zinc chloride	5	Purified to homogeneity	0.95	Azocoll	Wingard, Matsueda, and Wolfe (1972)
*A. mellea*	Fresh fruiting bodies; 1.5 kg	Ethanol	4	Purified to homogeneity	35	Azocoll	Lewis, Basford, and Walton (1978)
*G. frondosa*	Fresh fruiting bodies; 300 g	Ammonium sulfate	4	Purified to homogeneity	25	Azocasein	Nonaka et al. (1995)
*A. mellea*	Fresh fruiting bodies; 280 g	Ethanol	2	Purified to homogeneity	12	Azocasein	Healy et al. (1999)
*G. frondosa*	Fresh fruiting bodies; 23 g	Ammonium sulfate	2	Purified partially	31	hip-KV	Stressler et al. (2014)
*G. frondosa*	Dried fruiting bodies; 1.8 kg	20 mM Tris, pH 7.2	6	Purified to homogeneity	0.58	Azocasein	Zhao et al. (2020)

Even though commercial scale production of basidiomycetes like *G. frondosa* has been established (He et al. 2017), extraction and purification of PKMs from the fruiting bodies could present several challenges which can affect both the yield and efficiency of the process. As noted above, the fruiting bodies generally contain lower concentrations of PKMs (≤ 3%) compared to other sources, such as microbial cultures or recombinant systems. This would make it challenging to isolate PKMs in large quantities, potentially requiring the processing of large amounts of raw material to obtain a meaningful yield. This, in turn, would likely increase the cost of PKMs available for research. The fruiting bodies are composed of a complex mixture of proteins, polysaccharides, lipids, and other secondary metabolites. This introduces additional steps in the separation of the target PKM from other constituents, which can complicate purification and increase the risk of PKM degradation. The PKM content and activity within the fruiting bodies can vary due to environmental factors (temperature, humidity, etc.), cultivation practices (substrate composition, growth duration), and maturity stage of the fruiting bodies during harvest (Wu, Siu, and Geng 2021). This variability could lead to inconsistent PKM yields and quality, making large-scale production of research grade PKM less predictable.

### *Heterologous protein production methods*

The complete amino acid sequence of the PKM from *G. frondosa* is known (UniProt accession # P81054; Nonaka et al. 1997). Using UniProt’s basic local alignment search tool (BLAST), 250 proteins with variable sequence homology can be identified currently. Out of these 250 proteins, 9 proteins share ≥ 70% sequence homology and 104 proteins share greater than 50% but less than 70% sequence homology with *G. frondosa*’s PKM. To date, PKMs from only *G. frondosa*, *A. mellea*, and *T. coccinea* have been recombinantly expressed and described. This underscores the extensive diversity of unexplored PKMs that remain uncharacterized. The three PKMs share ≥ 60% sequence homology and were expressed in *K. phaffii* (Ahmed et al. 2024).

To prevent unintended proteolysis within the cell, endopeptidases are often produced recombinantly as inactive precursors, known as zymogens or pro-enzymes, which include inhibitory N-terminal pro-peptides (Demidyuk et al. 2010). Zymogen activation involves the cleavage and removal of the inhibitory pro-peptide, enabling proteolytic activity. This process requires recognition of a specific cleavage site, which can be targeted by an external peptidase (intermolecular activation) or by the zymogen’s catalytic domain (intramolecular activation) (Dumez et al. 2008). In some cases, the cleavage of pro-peptides occurs via proteolysis by pro-protein convertases in the host’s secretory pathway, yielding biologically active proteins. Protein folding and secretion are often the bottlenecks in heterologous protein expression. Even minor alterations in the pro-peptide sequence can significantly affect the expression and activity of the recombinant peptidase (Boon et al. 2020).

For producing heterologous proteins in *K. phaffii*, the secretion signal most frequently used is derived from the *Saccharomyces cerevisiae*’s α-mating factor pheromone. This signal consists of two distinct parts: an N-terminal sequence of 19 amino acids that directs protein entry into the endoplasmic reticulum (ER) and a 66-amino acid pro-region that aids in packaging into ER-derived COPII vesicles. In the ER, a signal peptidase removes the α-mating factor signal sequence, while the Kex2 peptidase in the Golgi cleaves the pro-peptide. This dual-function secretion signal has been effective for various heterologous proteins in *K. phaffii*, although secretion levels can vary significantly (Barrero et al. 2018). Kex2, also known as kexin (EC 3.4.21.61), was the first identified pro-protein convertase involved in the processing of α-mating factor and killer toxin precursors in *Saccharomyces cerevisiae*. Kex2 is also present in *K. phaffi* and preferentially hydrolyzes peptide bonds at the C-terminus of lysine-arginine (KR↓) and arginine-arginine (RR↓) residues present in the P1 position (Fuller et al. 1988).

The first report of a PKM expressed recombinantly was published by Saito et al. (2002). The researchers isolated cDNA of the PKM from *G. frondosa* by RT-PCR. The cDNA included the native pre-pro-protein sequence and a hexa-histidine tag at the C terminal. This synthetic cDNA was cloned into the *K. phaffi* expression vector pPIC3.5 under the control of the methanol-inducible promoter, “AOX1.” After 12 h of induction with 0.5% (v/v) methanol, three proteins with molecular weights of 41 kDa, 36 kDa, and 25 kDa were purified. The appearance of these three proteins was sequential. The authors concluded that the smaller proteins were products of autocatalysis of the 41-kDa protein. Caseinolytic activity was also detected sequentially with the appearance of the two smaller proteins. It was unclear if both or only one of the smaller proteins was proteolytically active. No proteolytic activity was observed with the 41-kDa protein. The study did not report the volumetric titer of the active PKM that was produced; however, it stated that 250 ng and 100 ng of the 36 kDa protein and 25 kDa protein were purified from an unspecified starting volume. The native pro-peptide sequence of the PKM from *G. frondosa* does not include a recognition site for Kex2 peptidase. This could explain the sequential appearance of the three distinct proteins during fermentation. *K. phaffii* may secrete a recombinant pro-protein rather than the mature protein due to incomplete processing of the signal peptide and/or pro-peptide within its secretory pathway.

Berger (2013) produced a mutant of PKM from *G. frondosa* which had the glutamic acid residue (E) at position 157 substituted with an aspartic acid residue (D). This site-directed mutagenesis resulted in altered cleavage specificity and the mutant PKM was able to recognize arginine (R) as well as lysine (K) present in the P1′ position as a cleavage site, allowing it to act as a sister enzyme to trypsin. The mutant PKM was expressed in both *E. coli* and *Baculovirus* expression systems; however, both strategies resulted in the production of PKM zymogen trapped inside inclusion bodies, thereby complicating the downstream processing.

The third report of a PKM expressed recombinantly was published almost 3 years later. Ødum et al. (2016) recombinantly produced native and mutant PKM from *A. mellea* in *E. coli* and *K. phaffii.* The attempts to produce enzymatically active PKM in *E. coli* using the native pro-protein and native mature protein sequences were unsuccessful on account of the formation of insoluble inclusion bodies. Three strategies were employed for expression in *K. phaffii.* The synthetic genes were expressed in *K. phaffi* under the control of the constitutive GAP promoter. All three constructs had the native signal peptide swapped with the signal peptide of *S. cerevisiae* OST1 and had a hexa-histidine tag added at the C-terminal. Construct #1 included the native pro-peptide sequence of the PKM. In construct #2, the native pro-peptide was swapped with the pro-peptide of α-mating factor of *S. cerevisiae.* In construct #3, the the native pro-peptide was linked to the pro-petide of α-mating factor of *S. cerevisiae*. Higher expression levels of recombinant PKM were observed with construct #1 vs construct #3. No commentary was provided on the expression levels of the PKM when construct #2 was utilized. A volumetric titer of 0.25 mg L^−1^ mature (active) PKM was obtained from the fermentation of construct #1. Unlike the recombinant PKM from *G. frondosa*, the wild-type recombinant PKM was recovered only in its mature (active) form via IMAC purification; the pro-enzyme was not detected on SDS-PAGE. This result appears to be in line with the fact that the native pro-peptide sequence (construct #1) contained a Kex2 peptidase recognition site (KR↓ and RR↓ present in P1 position) which would be targeted within the secretory pathway leading to the maturation of the PKM before it was secreted. Additionally, the researchers utilized the OST1 signal peptide compared to the native signal peptide used by Saito et al. (2002). OST1 has been shown to improve secretion of heterologous proteins in *K. phaffi* (Barrero et al. 2018)*.*

To assess whether or not the pro-PKM is capable of undergoing autocatalysis, the researchers induced mutations at the Kex2 recognition site. In mutant #1, the Kex2 recognition sites, “KR” and “RR,” were both changed to “QN.” In addition to these mutations, mutant #2 also had the catalytically active glutamic acid reside “E” replaced with asparagine “N.” Post purification, mutant #2 was detected as a single band of 36 kDa which corresponded to the MW of the pro-PKM, whereas mutant #1 was detected as, at least, 2 distinct bands of 19 kDa (corresponding to the mature PKM) and 15 kDa (corresponding to the released pro-peptide). Since the catalytically inactive mutant (#2) was only detected as a pro-protein in contrast to mutant #1 which appeared to undergo slow auto-activation, it was concluded that the main activation pathway of the PKM was still the cleavage at Kex2 recognition site between the pro-peptide and pro-enzyme.

Most recently, Ahmed et al. (2024) reported the production of the first known PKM with an acidic pH optimum from *Trametes coccinea.* The native pro-PKM attached to *S. cerevisiae*’s α-mating factor secretory signal was recombinantly expressed in *K. phaffii* under the control of the AOX1 promoter. The PKM was secreted into the culture broth both as pro-protein (38 kDa) and as active enzyme (19.8 kDa). The mature PKM was purified by anion exchange chromatography with a yield of 1.3 mg L^−1^. N-terminal sequencing using TMTpro Zero and mass spectrometry of the mature PKM indicated that the pro-peptide was cleaved between the amino acid positions 184 and 185 at the Kex2 cleavage site (KR↓) present in the native pro-protein sequence. The pro-PKM neither underwent auto-activation, nor was it processed by a trypsin-like endogenous *K. phaffi* peptidase. This result appears to be in line with Ødum et al.’s (2016) findings—with the notable exception that they isolated only the mature PKM from their culture medium. This could have been the result of *S. cerevisiae*’s α-mating factor being linked to the native pro-protein gene for *T. coccinea*’s PKM by a Kex2 cleavage site. The competition for cleavage at the two Kex2 sites (between α-mating factor and pro-protein and between pro-peptide and mature protein) could have resulted in limited maturation of the PKM zymogen resulting in the secretion of both inactive and active PKM.

The production of PKMs via recombinant protein engineering presents significant advantages over traditional extraction from natural sources, such as fruiting bodies of fungi like *Grifola frondosa*. While the use of fruiting bodies has historical relevance, modern biotechnology has the potential to outpace this method in terms of efficiency, scalability, and consistency. Recombinant protein production can ensure higher yields and scalability. The volumetric titer of the partially purified PKM by Stressler et al. (2014) was ≈3.52 mg L^−1^ after multiple rounds of precipitation, centrifugation, resuspension, and then eventual partial purification via FPLC. This titer was the highest among all the studies dealing with traditional production methods cited above. In comparison, Ahmed et al. (2024) produced ≈1.3 mg L^−1^ of pure recombinant PKM using shake-flask fermentation, demonstrating potential for further optimization and upscaling. The extraction of highly pure and active PKM from mushrooms presents challenges, including low yields, potential batch-to-batch variation, and resource-intensive processing. In contrast, recombinant expression in yeast systems such as *K. phaffii* could offer a more controlled and scalable alternative, potentially enabling more efficient PKM production.

For the recombinant production of PKMs, the choice of expression host depends on application scale and requirements for the enzyme. *K. phaffi* offers efficient secretion, proper folding, and scalability for active enzyme production, balancing cost, and activity. *Bacillus subtilis* and *Trichoderma reesei* are strong candidates for industrial-scale production due to their robust secretion capabilities, though endogenous proteolytic activity must be managed to prevent target protein degradation. In the two reported instances mentioned above, *E. coli* has been proven to be impractical due to the formation of inclusion bodies which required extensive downstream processing to obtain active PKM. Production in the baculovirus expression system attempted by Berger (2013) yielded similar results as *E. coli.* Although the capabilities of *K. phaffii* as a protein expression system are vast, there are certain limitations. In the context of PKMs, secretion of the active enzyme appears to be an area that needs to be optimized. Lower titers of mature PKMs can result from inefficient cleavage of the signal peptide, suboptimal pro-peptide cleavage due to a lack of necessary peptidases, or delays in proteolytic processing—especially under high protein secretion demands (Raschmanová et al. 2021). Inefficient protein secretion can have several causes in *K. phaffii*. Since the most frequently utilized ɑ-factor secretion signal directs post-translational translocation across the ER membrane, it may not work well for all proteins of interest (Zou et al. 2022). The nature of the signal sequence can control whether the protein travels via a co-translational or a post-translational pathway (Ng, Brown, and Walter 1996). With this knowledge, researchers can investigate different secretion signals to optimize the expression of PKMs. If translocation from the cytoplasm into the ER is an issue in protein secretion, then a different signal peptide such as OST1 could be utilized to resolve this issue and may drastically improve PKM secretion (Barrero et al. 2018). As the optimal choice of signal peptides is often protein specific, testing different signal peptides should influence overall yield. A protein-specific effect was demonstrated on the potential of secretion sequences by Karaoğlan (2023). Therefore, screening for more host signal peptides can offer potential alternatives to accomplish secretory expression of more heterologous PKMs.

Overexpression of heterologous proteins can lead to the saturation of its secretory pathway, elevating levels of unfolded proteins. Co-expressing chaperones in *K. phaffii* could enhance the secretion of PKMs by aiding in their proper folding and assembly within the endoplasmic reticulum (ER). Chaperones, such as BiP and protein disulfide isomerase (PDI), have been shown to help in preventing protein misfolding and aggregation, which would otherwise lead to ER stress and limit secretion (Robinson et al. 1996). By stabilizing newly synthesized PKMs, chaperones could improve protein quality control and facilitate the efficient transit of properly folded PKMs through the secretory pathway, ultimately increasing the yield of correctly folded, functional recombinant PKMs. Recently, co-expression of novel folding factors, such as Mpd1p, Pdi2p, and Sil1p, was shown to exhibit protein-specific effects on cell growth, transcription, and expression of different reporter genes (Duan et al. 2019). Strategies like optimizing the signal peptide, incorporating native *K. phaffii* processing sites, co-expressing required peptidases, or using tailored *K. phaffii* strains could be effective in achieving higher titers of novel PKMs.

### Applications of peptidyl-lys metalloendopeptidase (PKM): historical perspective and current developments

Due to PKM’s cleavage specificity for peptide bonds at the N-terminus of lysine residue (P1′ position), which complements the cleavage pattern of LysC, PKM was identified early on as a potential tool for proteomics applications, particularly as a sister enzyme to LysC. However, it was not until 2005 that the first definitive application of a PKM in the field of proteomics was reported (Rao, Carruth, and Miyagi 2005). An extensive search of currently available literature indicates that the application of PKMs continues to stay limited to the field of proteomics. The selective cleavage of lysine residue present in the P1′ position is particularly valuable in mass spectrometry (MS)–based proteomics, where it enables consistent peptide fragmentation patterns, facilitating peptide mapping and protein identification. Outside of proteomics, however, its high specificity is less versatile, potentially limiting its utility in broader applications, such as general protein hydrolysis or industrial bioprocessing, where broader specificity is often required for efficient substrate breakdown.

### *Proteomics*

In recent decades, shotgun proteomics, also referred to as bottom-up proteomics, has emerged as a cornerstone of mass spectrometry (MS)–based proteome analysis. This growth is attributed to advancements in every step of the proteomics workflow, including cell lysis, peptide separation, fragmentation, MS instrumentation, and database search algorithms. Despite these innovations, protein digestion continues to rely predominantly on a single enzyme: trypsin (Jiang et al. 2024). Trypsin, a ~ 23 kDa serine peptidase, is essential in proteomics for breaking down proteins into smaller peptides. It cleaves peptide bonds on the carboxyl side of lysine and arginine residues present in the P1 position, functioning optimally in a pH range of 7.0–8.5 (Olsen, Ong, and Mann 2004). At acidic pH (< 6), trypsin loses activity and may denature, while highly alkaline conditions (pH > 9) also reduce its efficiency (Dau, Bartolomucci, and Rappsilber 2020). PKM on the other hand can maintain activity over a broader pH range (4.0–10.0; Ahmed et al. 2024). PKMs have emerged as crucial tools in proteomics for their ability to perform targeted and specific cleavage at lysine residues present in the P1′ position. In proteomics, the choice of peptidase significantly affects the efficiency, specificity, and outcome of MS-based protein identification (Tsiatsiani and Heck 2015). While trypsin remains the gold standard, alternative enzymes, such as PKM and LysC, have been introduced to expand peptide coverage and improve analytical outcomes. Unlike trypsin, which cleaves at the C-termini of both lysine and arginine residues, PKM produces unique cleavage patterns, offering complementary information that expands protein sequence coverage and enhances data reliability in complex sample analysis (Taouatas, Heck, and Mohammed 2010). The N-terminus cleavage pattern generated by PKM differs from the C-terminus cleavage pattern produced by trypsin and LysC, resulting in complementary peptide fragments. When used in tandem with trypsin and LysC, PKMs can enhance sequence coverage, improving the detection of peptides that trypsin alone might miss (Dau, Bartolomucci, and Rappsilber 2020). PKM primarily cleaves at the N-terminus of lysine, but missed cleavages occur in some cases. In yeast data, 61% of peptides had expected cleavages at both termini, 36% at only one, and 3% at neither. Nonspecific cleavages often occurred next to alanine, serine, or arginine (~ 70% of cases), suggesting a preference rather than random errors (Hohmann et al. 2009). Ahmed et al. (2024) reported high sequence coverage (84%) of BSA hydrolyzed with the PKM from *T. coccinea* whereas comparable sequence coverage of BSA (90%) was observed when trypsin was used as peptidase in a control experiment. However, a higher number of unique peptides was observed when trypsin was used.

Trypsin typically generates smaller peptides, providing good sequence coverage; however, the spacing of arginine and lysine residues acids within the protein sequences influences the length of peptides, and thus the number of unique peptides with molecular masses is large enough to be detected via MS. PKM cleaves less frequently, potentially producing longer peptides that provide better coverage of readily detectable peptides, complementing trypsin’s limitations. Table 3 illustrates this by depicting the different cleavage pattern obtained after digesting Histone H3 (Uniprot ID B2R6Y1) with trypsin and a representative PKM. While digestion by PKM generates 4 peptides and one single amino acid with molecular masses < 500 Da, digestion by trypsin generates 11 peptides and 8 single amino acids with molecular masses < 500 Da. Although PKM-generated peptides may be longer and less optimal for ionization, they perform well in tandem MS, especially when used in combination with trypsin (Hohmann et al. 2009).

**Table 3:** Peptide fragmentation pattern resulting from the digestion of Histone H3 by PKM and trypsin

	**Digestion of Histone H3 by PKM**		
**# of cleavage sites**	**Position of cleavage site**	**Peptide sequence**	**Peptide mass [Da]**
12	4	MART	478
	9	KQTAR	603
	14	KSTGG	448
	18	KAPR	471
	23	KQLAT	560
	27	KAAR	445
	36	KSAPSTGGV	803
	37	K	146
	56	KPHRYRPGTVALREIRRYQ	2397
	64	KSTELLIR	959
	79	KLPFQRLVREIAQDF	1860
	115	KTDLRFQSAAIGALQEASEAYLVGLFEDTNLCAIHA	3867
	136	KRVTIMPNDIQLARRIRGERA	2494
	**Digestion of Histone H3 by trypsin**		
**# of cleavage sites**	**Position of cleavage site**	**Peptide sequence**	**Peptide mass [Da]**
28	3	MAR	376
	5	TK	247
	9	QTAR	475
	10	K	146
	15	STGGK	448
	18	APR	342
	19	K	146
	24	QLATK	560
	27	AAR	316
	28	K	146
	37	SAPSTGGVK	803
	41	KPHR	537
	50	YRPGTVALR	1032
	53	EIR	416
	54	R	174
	57	YQK	437
	64	STELLIR	831
	65	K	146
	70	LPFQR	660
	73	LVR	386
	80	EIAQDFK	850
	84	TDLR	504
	116	FQSAAIGALQEASEAYLVGLFEDTNLCAIHAK	3382
	117	R	174
	129	VTIMPNDIQLAR	1371
	130	R	174
	132	IR	287
	135	GER	360
	136	A	89

Trypsin-generated peptides are typically positively charged at the C-terminus, which enhances their ionization and detection in electrospray ionization (ESI) and tandem MS. In contrast, PKM produces peptides with an N-terminal lysine, altering the charge distribution and fragmentation pattern. This difference can improve MS coverage, especially for peptides that might escape detection with trypsin alone (Meyer and Komives 2012). Although trypsin remains the gold standard in proteomics workflows, PKMs offer complementary benefits. Cleavage at the N-terminus of lysine provides additional sequence coverage, particularly for proteins with lysine-rich domains or post-translational modifications (PTMs) involving lysine. PKMs are especially useful for de novo sequencing or analyzing specific modifications at lysine residues, where they can target regions that trypsin might overlook (Boersema et al. 2009). PKMs are capable of cleaving adjacent to mono- and dimethylated lysine residues, offering advantages in detecting lysine-specific PTMs (Hohmann et al. 2009). While trypsin’s broad cleavage pattern is effective for many applications, it may limit the precise identification of lysine-specific PTMs, such as ubiquitination, acetylation, or methylation, as modified sites may be missed. Recently, the use of the PKM enabled five-plex quantification at both stages of mass spectrometry analyses, i.e., MS1 and MS2 levels, was demonstrated. This multiplexing capability allowed for the inclusion of a reference channel, which significantly enhanced the proteomic depth achievable in single-cell analyses. Consequently, the workflow facilitated the routine analysis of up to 80 single cells per day, thereby improving throughput and robustness in single-cell proteomic (Thielert et al. 2023). Increased basicity at the amino terminus also strengthens the b-ion series, generating extended b-ion ladders that enhance database search algorithms (Hohmann et al. 2009). PKM can also be used in combination with other peptidases besides trypsin. To enhance the identification of C-terminal peptides, PKMs, alongside the recently introduced LysargiNase (Huesgen et al. 2014; Tallant et al. 2006), offer an alternative for generating positively charged C-terminal peptides compatible with LC-MS/MS analysis. PKM also functions as a complementary enzyme to lysyl endopeptidase (LysC), which selectively hydrolyzes lysine residues present in the P1 position (Kishimoto et al. 2011; Zhao et al. 2020). Combining PKM with LysC in digestion workflows enables the production of a wider variety of unique peptide fragments, enhancing protein identification and the mapping of post-translational modifications. PKM and LysC also demonstrate superior tolerance to extreme denaturing conditions, including high and low pH, elevated temperatures, organic solvents (e.g., acetonitrile), and up to 8 M urea, making them well suited for challenging protein samples. These properties have facilitated the development of novel proteomic strategies, such as combining PKM and LysC digestions to precisely distinguish protein N- and C-termini in a single experiment, without requiring additional labeling or enrichment steps (Kishimoto et al. 2011).

Trypsin, LysC, and the PKM peptidases known until very recently are only effective between neutral to alkaline pH range. In disulfide mapping experiments, the alkaline conditions optimized for trypsin and LysC digestion can cause disulfide bond rearrangement (Sanger 1953). This issue can be addressed by conducting digestion at suboptimal pH levels or using a peptidase that is effective under acidic conditions. The broader operational pH range of the PKM from *T. coccinea* could enable the exploration of acidic buffer systems for MS-based proteomics applications. For example, this PKM could be ideally suited for disulfide mapping experiments due to its acidic pH optimum at pH 5.0. Sample digestion at pH 5.0 prevents disulfide exchange reactions that usually take place at alkaline pH. Avoiding disulfide exchange reactions might lead to a lower number of false positive identified disulfide bridges.

### Outlook

Enzyme engineering holds promise for expanding the application of PKMs in synthetic biology. Recent in silico digestion of honey samples by PKMs revealed peptides with potential anti-oxidant, anti-microbial, and anti-diabetic properties (Alarjani and Mohammed 2024). Lowering the cost of commercially available PKMs will be key to making them more accessible to researchers globally. Currently, the price of recombinant PKM is ≈12 times higher than that of recombinant trypsin. As new PKMs from diverse sources are discovered, produced, and made available at affordable prices, their use could extend into therapeutic and food applications, where they might generate specific functional peptides. Recombinant production systems allow protein engineering to potentially alter characteristics of PKMs if needed. This precision is not possible when extracting PKMs from natural sources, where no control exists over the enzyme’s genetic or structural variability. With protein engineering, optimizing PKM's cleavage specificity, stability, and activity under different experimental conditions could allow the design of tailored variants for specific biochemical reactions. Future research may also focus on developing fusion enzymes with broader or more precise cleavage capabilities, enabling controlled digestion of complex substrates like therapeutic proteins or synthetic peptides used in drug delivery systems (Wang et al. 2022).

## Data Availability

Not applicable.
